# Defining usual physiotherapy care in ambulant children with cerebral palsy in the United Kingdom: A mixed methods consensus study

**DOI:** 10.1111/cch.12977

**Published:** 2022-02-11

**Authors:** Rachel Rapson, Jos M. Latour, Jonathan Marsden, Harriet Hughes, Bernie Carter

**Affiliations:** ^1^ Physiotherapy Torbay and South Devon NHS Foundation Trust Torquay UK; ^2^ School of Nursing & Midwifery, Faculty of Health and Human Sciences University of Plymouth Plymouth UK; ^3^ Plymouth Hospitals NHS Trusts Plymouth UK; ^4^ Musgrove Park Hospital Taunton UK; ^5^ School of Health Professions University of Plymouth Plymouth UK; ^6^ Torbay and South Devon NHS Foundation Trust Torquay UK; ^7^ Faculty of Health and Social Care Edge Hill University Ormskirk UK

**Keywords:** cerebral palsy, consensus, nominal group, physiotherapy, walking

## Abstract

**Background:**

Ambulant children with cerebral palsy (CP) undertake physiotherapy to improve balance and walking. However, there are no relevant clinical guidelines to standardize usual physiotherapy care in the United Kingdom. A consensus process can be used to define usual physiotherapy care for children with CP. The resulting usual care checklist can support the development of clinical guidelines and be used to measure fidelity to usual care in the control groups of trials for children with CP.

**Methods:**

Twelve expert physiotherapists were recruited. In Phase 1, statements on usual care were developed using a survey and two nominal groups. Phase 2 included a literature review to support usual physiotherapy interventions. Phase 3 used a confirmatory survey, which also captured changes to provision during the COVID‐19 pandemic. Consensus was calculated by deriving the mean of the deviations from the median score (MDM). High consensus was deemed to be where MDM < 0.42.

**Results:**

Physiotherapists reached high consensus on five outcome measures (MDM range 0–0.375) and nine areas of assessment (MDM range 0–0.25). Physiotherapists reached moderate consensus on task‐specific training (MDM = 0.75), delivered at weekly intensity for 4–6 weeks (MDM = 0.43). There was high consensus (MDM = 0) that children should participate in modified sport and fitness activities and that children with Gross Motor Function Classification System Level III should be monitored on long‐term pathways (MDM = 0.29).

**Conclusions:**

Physiotherapists reached consensus on two usual care interventions, and a checklist was developed to inform the control groups of future randomized controlled trials. Further consensus work is required to establish clinical guidelines to standardize usual physiotherapy care in the United Kingdom.

**Key messages**

- A checklist of usual physiotherapy care in the United Kingdom has been developed for ambulant children with cerebral palsy to inform the control groups in randomized controlled trials.
- Usual physiotherapy care should include task‐focused therapy, facilitation of modified sport and participation in community activity.
- Physiotherapy tools were identified for the assessment of balance and mobility and measurement of treatment outcomes.
- Children with Gross Motor Function Classification System Level III should remain on long‐term monitoring pathways.
- The usual intensity of physiotherapy treatment in the United Kingdom is weekly for 4–6 weeks and is lower than that which is reported to be effective in research literature.

## 1 INTRODUCTION

Cerebral palsy (CP) is an umbrella term describing a group of permanent disorders affecting the development of posture and movement affecting 2.1 per 1000 children (Oskoui et al., 2013). Motor impairments associated with CP make walking more effortful and significantly limit children's participation at school and in the community (Kamp et al., 2014). Children with CP can experience primary movement impairments such as spasticity, weakness or reduced selective movement control (Rosenbaum et al., 2007). The severity of the movement disorder can be described using the Gross Motor Function Classification System (GMFCS) (Palisano et al., 1997). Children with GMFCS Levels I–III are able to walk with varying levels of support or orthoses and tend to achieve their peak motor performance by age 9 (Hanna et al., 2008; Palisano et al., 2007). However, secondary musculoskeletal impairments can develop during periods of rapid growth, presenting further challenges to walking and balance skills.

Physiotherapists provide advice and therapeutic interventions aimed at addressing primary impairments and preventing secondary complications of CP. Young people with CP and their families want to know which physiotherapy interventions are the most effective and the frequency and intensity required to achieve optimum mobility (Morris et al., 2015). Physiotherapy service provision may vary depending on resources and how emerging evidence (Franki et al., 2012; Hägglund et al., 2014; Novak et al., 2013) and national guidance is implemented (Mugglestone et al., 2012). Currently, there is no standardization of physiotherapy care for ambulant children with CP in the United Kingdom.

The highest level of evidence for the effectiveness of an intervention is through meta‐analysis of randomized controlled trials (RCTs) (Centre for Evidence‐Based Medicine, OCEBM Levels of Evidence Working Group, 2011). In many physiotherapy studies, the control group undertakes ‘usual care’, but this is often unspecified. Usual care across studies is likely to vary in the frequency and intensity of physiotherapy, and participants in a control group could be undertaking activities similar to the experimental intervention. It is essential to define usual care within the research setting to ensure the effect size of an intervention within a trial is correctly measured. Therefore, a definition of usual care is crucial to ensure robust research findings and to inform the development of evidence‐based clinical pathways (Royal College of Physicians, 2016).

## 2 METHODS

The aim of this study was to reach consensus on current usual physiotherapy care delivered by physiotherapists in the United Kingdom and to develop a usual care checklist to enable measurement of fidelity to usual care in the control group of a forthcoming feasibility RCT. The Health Research Authority and Health and Care Research Wales (reference 254056) granted permission for this study.

**FIGURE 1:**
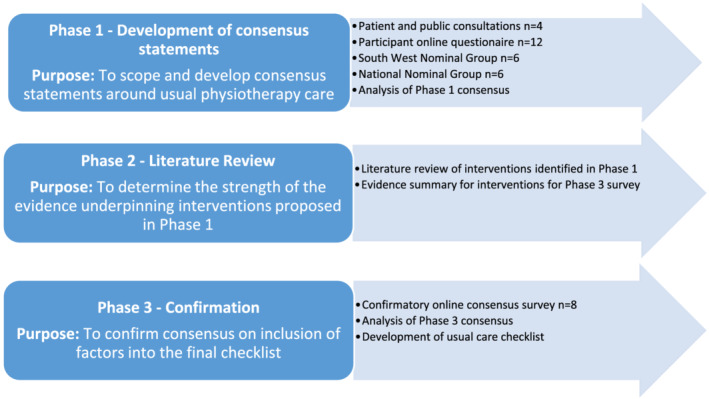
Flow diagram showing the three phases of the consensus study

This study adopted a three‐phase design (Figure 1). Phase 1 used idea generation and nominal group technique (NGT) to establish consensus statements on usual physiotherapy care aimed at improving balance and walking in children with CP, GMFCS I–III. Phase 2 was a literature review to establish the evidence base underpinning the interventions identified in the consensus statements. Phase 3 used a survey to confirm consensus on the usual care checklist.

### 2.1 Participants

The optimal size for a nominal group (NG) is between 5 and 12 people (Allen et al., 2004; Harvey & Holmes, 2012; Potter et al., 2004). Two NGs were established in Phase 1. The first NG consisted of six paediatric community physiotherapists from National Health Service (NHS) providers in South West UK. The physiotherapy managers of five child development centres recruited participants. They gave information packs to interested clinicians. The manager was asked to nominate one or two staff volunteers to participate during work time. A national NG was formed with six community physiotherapists from the rest of the United Kingdom. Adverts were placed in the Association of Paediatric Chartered Physiotherapists e‐bulletin. Interested physiotherapists were invited to respond directly to the chief investigator, who sent them an information pack. Participants were eligible if they had over 2 years of experience in paediatric physiotherapy and held a current community paediatric caseload in the United Kingdom, with an NHS provider. In Phase 3, all 12 participants from Phase 1 were invited to complete a confirmatory survey.

### 2.2 Phase 1: Development of consensus statements

Phase 1 employed the NGT, a consensus process that encourages individual participation and a non‐hierarchical exchange of ideas (Ven & Delbecq, 1974). It has previously been used within physiotherapy to reach consensus on interventions that influence motor development in children with CP (Bartlett & Palisano, 2002). NGT involves a three‐stage process of decision‐making during a structured group meeting led by a skilled, neutral facilitator (Bartlett & Palisano, 2002; Delbecq et al., 1975).

#### 2.2.1 Idea generation

We modified the NGT by using an online questionnaire to develop ideas prior to the NG meetings. In addition to the questionnaire, participants received a clinical scenario, describing a 12‐year‐old boy with CP (GMFCS Level II), to help them frame their responses using an authentic situation. The questionnaire comprised a series of open questions to explore ideas on what constitutes usual physiotherapy care for him and how it might vary for children of different ages and functional levels. The lead author grouped together the responses generated by participants to form 10 statements about usual care. Ideas excluded from the 10 statements, where fewer than 20% respondents identified them, were recorded and set aside for discussion and clarification during the NGs.

#### 2.2.2 NGs

The lead author, an experienced paediatric physiotherapist and researcher, facilitated the NGs. Her position at the group was of a neutral facilitator, and other members of the research team supported the process: HH documented notes, and JM administered the scoring. Participants were asked to consider the minimal physiotherapy care usually undertaken by a physiotherapist, regardless of NHS setting. Careful consideration was given to the scope of the physiotherapy role. Participants excluded the provision of orthotics, as orthotists are autonomous practitioners responsible for the assessment and prescription of orthotics.

The statements on usual care were presented to participants at the beginning of the SW NG. Participants scored their level of agreement with each statement using a 5‐point Likert‐type scale (1 = *strongly disagree*, 2 = *disagree*, 3 = *undecided*, 4 = *agree* and 5 = *strongly agree*). The mean group score was calculated for each statement at the end of each scoring round. Participants were presented with the group median score alongside their individual scores for each statement. The facilitator encouraged a round‐robin feedback from the participants for each statement. Participants explored the relative merits of each statement and were able to evaluate their ideas compared with those held by the group. Participants discussed and then revised the statements. The group revisited any ideas previously set aside for further discussion to see if they wished to include them. For example, hydrotherapy was a subject initially set aside, and was revisited by both groups, but remained excluded. Participants rescored all the statements where consensus was not reached in the previous scoring round.

The statements on usual care developed during the South West (SW) NG were presented at the beginning of the national NG, in an iterative process. The national NG decided to include an idea that had been excluded by the SW NG. This was related to the importance of advocating wheelchair mobility for children assessed as GMFCS Level III. This was taken forward into Phase 2.

At the end of Phase 1, the levels of consensus for the 10 statements on usual physiotherapy care were calculated for each NG. Six physiotherapy interventions were proposed by the NGs as usual care.

### 2.3 Phase 2: Literature review

The aim of the literature review was to appraise the strength of evidence supporting the six interventions proposed for inclusion (in Phase 1) in usual care for ambulant children with CP.

#### 2.3.1 Search strategy

Two researchers (RR and JM) conducted the search for literature systematically. No date limits were set for the search. The initial search took place on 16 December 2019 and was updated as new evidence emerged until 07 July 2020. The databases searched were MEDLINE (EBSCO), EMBASE (EBSCO), PUBMED, The Cochrane Central Register of Controlled Trials, CINAHL, AMED (EBSCO), PEDro, SCOPUS, Google Scholar, ETHOS, PRIMO research outputs and theses.

Initial keywords searched were child OR adolescent AND cerebral palsy AND physiotherapy OR physical therapy AND walking OR gait OR balance AND strength training OR exercise OR progressive resisted exercise OR strengthening OR stretching OR flexibility OR task practice NOT surgical OR Botulinum toxin OR orthotic OR orthoses.

#### 2.3.2 Inclusion and exclusion criteria and study selection

Systematic reviews or studies in the English language were included where they reported physiotherapy interventions with outcomes related to walking and balance. Where no systematic review was found, RCTs and then experimental studies were included. Papers were excluded where the results are reported in a systematic review or were superseded by more recent studies. Protocol‐only publications and papers that did not report an outcome relating to balance or walking were excluded. The results are presented in accordance with PRISMA guidelines (Moher et al., 2011).

**FIGURE 2:**
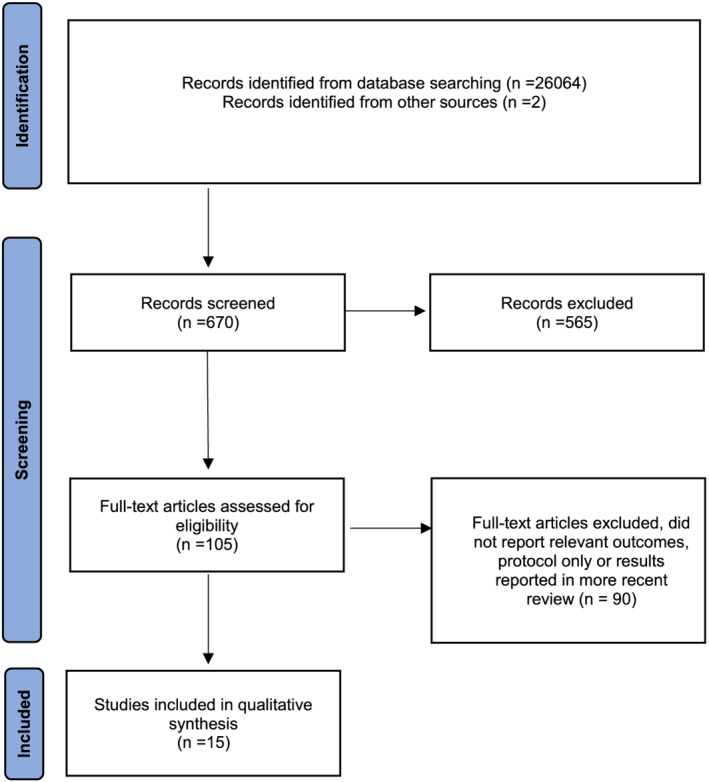
PRISMA diagram showing the flow of citations reviewed within the literature review

Figure 2 shows that of the 670 abstracts reviewed, 105 full papers were retrieved for abstract review; of these, there were 75 systematic reviews, 29 RCTs and one experimental design study. Only 15 papers met the criteria for full review and were assessed for bias using the CASP tool (Hannes et al., 2010). These comprised 12 systematic reviews (Anttila et al., 2008; Bania et al., 2019; Booth et al., 2018; Clutterbuck et al., 2019; Corsi et al., 2021; Dewar et al., 2015; Elnahhas et al., 2019; Franki et al., 2012; Galey et al., 2017; Moreau et al., 2016; Novak et al., 2020; Ritzmann et al., 2018; Tustin & Patel, 2017), two RCTs (Kimoto et al., 2019; Valentín‐Gudiol et al., 2017) and one non‐randomized crossover trial (Salem et al., 2010). The strength of evidence for interventions identified as usual care were rated as high, moderate, low or very low levels of evidence (Balshem et al., 2011) (see Table 1).

**TABLE 1:** Evidence summary for physiotherapy interventions aimed at improving walking and balance for children with cerebral palsy

Intervention	Evidence the intervention improves balance or walking	Evidence strength	Reference
Participation in physical activities	Aerobic and fitness training improves gross motor function	Moderate	Clutterbuck et al. (2018)
	Modified sport improves balance and walking	Low	Clutterbuck et al. (2018)
Flexibility exercise	No evidence found	Very low	
Prolonged passive stretching	Serial casting of the ankle improves in gait parameters in the short term (<12‐week effect), but it is unclear whether there is functional benefit	Low	Tustin and Patel (2017)
	Serial casting does not improve stride length	Very low	Corsi et al. (2019)
	Prolonged standing in a frame or tilt table for 45 min, 3 times a week may have a short‐term, positive effect on gait parameters	Low	Salem et al. (2010)
Strength training	Strength training using progressive resisted exercise does not improve gross motor function, gait speed and gait characteristics	High	Clutterbuck et al. (2018); Corsi et al. (2019); Dewar et al. (2015)
	Progressive resisted exercise does not improve postural control in standing	Moderate	Dewar et al. (2015)
	Gross motor activity training with progressive resisted training (e.g. loaded sit to stand) does not improve gross motor function and is associated with multiple adverse events	Moderate	Clutterbuck et al. (2018)
Task‐specific training and functional activity training	Gross motor activity training improves gross motor function when undertaken in real‐world situations with variable practice of skills	Moderate	Bania et al. (2019); Clutterbuck et al. (2018)
	Gross motor task training of 1 h, 2–5 times per week for 5–6 weeks improves postural stability during gait	Moderate	Dewar et al. (2015)
	Mobility training, treadmill training and partial body‐weight support treadmill training increases walking and stride length at a dose of 15–30 min, 2–7 times per week for 6–7 weeks	Moderate	Bania et al. (2019); Booth et al. (2018); Clutterbuck et al. (2018); Corsi et al. (2019); Novak et al. (2020)
	Treadmill training (excluding partial body weight supported) improves balance and postural control	Moderate	Dewar et al. (2015)
	Backward gait training improves balance, gross motor function, step length and walking velocity at a dose of 15–25 min, 3 times per week for 6–12 weeks	Moderate	Elnahhas et al. (2019)
	Partial body‐weight support treadmill training improves gross motor function and walking endurance	Low	Novak et al. (2020)
Postural stability and balance activities	Full body vibration training improves gait speed at a dose of 9–18 minutes, 3 times per week for 8 weeks	High	Corsi et al. (2019)
	Trunk training on vibration plate improves trunk alignment during gait	Moderate	Dewar et al. (2015)
	Neurodevelopmental therapy for 30 min twice a week for 8 weeks did not improve standing balance in children with spastic diplegia	Low	Dewar et al. (2015)

### 2.4 Phase 3: Confirmatory survey

The final online survey allowed participants to score subsections of each statement of usual care in more detail. For example, participants were asked to rate individual assessment tools from the list identified in Phase 1 using the 5‐point Likert‐type scale. Interventions were presented alongside the evidence summary (Table 1), and participants were asked to indicate whether they thought the intervention should be included or excluded as usual care or if they were undecided. Participants were asked to comment on why they decided to award each score in order to gain more insight into their views and experiences.

### 2.5 Analysis

Consensus was calculated by deriving the mean of the deviations from the median score (MDM) using the following equation (Gunn et al., 2018):

$$\frac{\mathrm{MDM}=\mathrm{sum}\;\text{of individual deviations from the median}}{\text{number of participants}}$$

High consensus (MDM < 0.42) is required for any treatment intervention to be considered important for inclusion, for example, type of exercise, whereas moderate consensus (MDM = 0.42–0.81) is acceptable for other aspects of the programme setting such as method of delivery (Allen et al., 2004).

Text from the idea generation questionnaire, quotations noted during the NGs and responses from the confirmatory survey were transcribed and coded as follows: P representing participant, followed by participant number and either NG = nominal group or S = survey to show at which stage it was said. The confirmatory survey produced anonymous responses from individuals representing both NGs. The text was explored using a framework analysis approach.

## 3 RESULTS

Twelve physiotherapists participated across the two NGs in Phase 1. The median age of participants was 43 years (range 28–60) with a median level post qualification of 21.5 years (range 7–38) with 18.5 years (range 3–29) in paediatrics. Table 2 shows the similarity between both NGs. Eight of the 12 participants completed the Phase 3 confirmatory anonymous survey.

**TABLE 2:** Mean age, location and experience of participants

	All participants *n* = 12	South West NG *n* = 6	National NG *n* = 6
Median participant age (range) years	43 (28–60)	40 (28–60)	45 (31–59)
Median number years (range) qualified as a physiotherapist	21.5 (7–38)	18 (7–39)	23 (7–38)
Median number years (range) working in paediatrics	18.5 (3–29)	15 (3–29)	20.5 (7–25)
Location of NHS Providers represented		Plymouth, Exeter, Torquay, Truro	Chelmsford, Kent, Leicester, London, Medway, Yorkshire

Abbreviations: N, number; NG, nominal group.

Participants developed 10 statements on usual care during the NGs. They described six areas of intervention to be included in the literature review: participation in physical activities, flexibility exercises, prolonged passive stretching; strength training; and task‐specific or functional activity training. Participants identified a list of assessment tools and outcome measures to be included in the confirmatory survey. Both groups reached a high level of consensus (MDM < 0.42) for all 10 statements on usual care at the end of the Phase 1 process (Table 3). Participants in the SW group tended to award a higher median score for each topic.

**TABLE 3:** The level of consensus scoring of statements of usual care in Phase 1

Statement topic	SW group		National group		
	Median score	MDM	Median score	MDM	Level of Consensus
Referral and discharge	5	0.25	4	0.17	High
Location of therapy	4.5	0.38	5	0	High
Frequency and intensity	5	0.25	4.5	0	High
Advice and information	5	0	5	0.33	High
Goals setting	5	0.5	5	0.33	High
Assessment tools	5	0.25	4.5	0	High
Outcome measures	5	0.25	5	0.5	High
Interventions	5	0	4.5	0	High
When frequency and intensity of physiotherapy differs	5	0.25	5	0.33	High
How intervention differs in relation to GMFCS level	5	0	4	0.33	High
How outcome measure differs in relation to GMFCS level	5	0	5	0.5	High
How intervention differs in relation to the child's age	5	0	4.5	0	High
How outcome measure differs in relation to the child's age	5	0	5	0.5	High

Abbreviations: GMFCS, Gross Motor Function Classification Scale; MDM, mean deviation from median; SW, South West.

The literature review appraised evidence for the six interventions identified as usual care during Phase 1. Evidence for each intervention was explored in relation to outcomes of walking, balance and gross motor function. The evidence summary (Table 1) shows moderate to low evidence to support fitness training and modified sport. There was low evidence supporting prolonged passive stretching (excluding orthotics) using serial casting or prolonged standing frame use. There was moderate to high evidence against the use of progressive strength training. Strength training did not improve gait characteristics or postural control and was associated with multiple adverse events. Task‐specific training, focusing on gait training on the treadmill or on the ground, was supported by a large evidence base, with low to moderate evidence supporting its use. There was moderate to high evidence supporting the use of vibration plate training for postural stability and improving gait and low evidence against the use of neurodevelopmental therapy for standing balance. There was an absence of literature to support flexibility, postural stability or balance exercises as described by participants.

The results below amalgamate the consensus responses with the results of the literature review. Results are presented under two main themes: physiotherapy service provision and structure and physiotherapy interventions. Consensus scores are presented for each statement topic alongside direct quotations from the participants. Where a view was sustained from Phase 1, this is documented to show how the view was developed.

### 3.1 Physiotherapy service provision and structure

#### 3.1.1 Referral and discharge criteria

There was high consensus (MDM = 0.29) that children with GMFCS Level III should remain on a long‐term pathway, from initial referral until they transition to adult services. This view was sustained from Phase 1 to Phase 3, for example:

Children with GMFCS III are more likely to develop joint contractures and muscle shortening affecting function …. They have on going equipment needs. (P4‐NG)

The pathway should include monitoring schedules for range of motion and hip surveillance, such as the Cerebral Palsy Integrated Pathway (CPIP), and continue until skeletal maturity (Wordie et al., 2020). There was high consensus (MDM = 0.14) that children at GMFCS Levels I and II require episodes of care related to individual need as P8 explains:

They may also run into difficulties around growth spurts but can be given red flag information for re‐referral. (P8‐NG)

Participants supported the prioritization of early intervention in younger or newly diagnosed children.

#### 3.1.2 Location of physiotherapy appointments

High consensus established that usual care takes place in a children's outpatient clinic (MDM = 0) and that appointments occur at school or home (MDM = 0.14) when there are equipment or environmental needs. This is often due to post‐surgical rehabilitation programmes or comorbidities such as learning disability, where treating the child in the context of their usual environment is deemed to be more effective. Physiotherapists frequently visit schools to train support workers to deliver a delegated programme of usual physiotherapy care. Time efficiency was a factor affecting this choice:

It is … more time‐efficient to see children in the department. However, we carry out home or school visits if indicated to review equipment or specific activities related to school or home environment. (P3‐S)

#### 3.1.3 Frequency and intensity of physiotherapy input

There was high consensus (MDM = 0) that the clinical needs of the children dictate the frequency and intensity of blocks of treatment and reviews. There was moderate agreement (MDM = 0.43) that blocks occur once per week for 4–6 weeks. This was first identified in Phase 1 and sustained in Phase 3:

4–6 treatments appear to be what is manageable for children and their families to follow a more demanding therapy regime. It allows for review of goals and monitor[ing] progress in a defined timespan. (P11‐NG)

There was high consensus (MDM = 0) that children receiving physiotherapy should be routinely reviewed every 3–12 months. There was high consensus (MDM = 0.25) that physiotherapy is needed more often in early years and especially during transition to nursery, school and adult services. Physiotherapy support may be required more frequently when parents have additional needs, such as learning disabilities.

There was high consensus (MDM = 0) that intensive blocks of physiotherapy rehabilitation are indicated following procedures (e.g. botulinum toxin injections, orthopaedic surgery and serial casting), during growth spurts and where there are changes in spasticity medications or orthotic provision. There was high consensus (MDM = 0.38) that rehabilitation after selective dorsal rhizotomy (SDR) surgery requires a highly intense period of rehabilitation, several times per week over 12 or more months (and requires a specific funding package).

#### 3.1.4 Advice, training and information

There was high consensus (MDM = 0.29) that physiotherapists play an important role in supporting children and their families to understand the impact of their diagnosis and the prognosis of their condition. Participants reached high consensus (MDM = 0.29) on the importance of sharing information across agencies, where parents and children give their consent. This typically includes information in the form reports and Education and Health Care Plans (EHCPs) (Tutt & Williams, 2015) and training for parents and teaching staff who deliver the child's therapy programme. Physiotherapists also provide information regarding local and national resources, such as the statutory local offer, charitable organizations and support groups. The group emphasized the value of this, with a typical response being:

We could do more to educate wider school staff and potentially other pupils to help them understand the condition and how it effects an individual. (P3‐S)s

#### 3.1.5 Goal setting

There was high consensus (MDM = 0.25) that physiotherapists use the Specific Measurable Achievable Realistic Timed (SMART) goal setting approach. Participants emphasized the need to set goals collaboratively, at the level of participation rather than body structure and function (World Health Organization, 2007):

A goal needs to be meaningful to the child/family rather than medical. It can quite often be challenging to make a meaningful goal out of a medical need e.g. better heel strike may be achieved and step length improved but the family struggle to see a functional benefit and we do not spend enough time exploring what this gain means to them in terms of their life demands. (P2‐S)

#### 3.1.6 Assessment

Participants identified 11 areas of assessment of mobility and balance in Phase 1. In Phase 3, participants reached high consensus (MDM range 0–0.25) for nine areas of assessment covering function, range of movement (ROM), muscle tone, gait, posture and pain (Table 4).

**TABLE 4:** The level of consensus on assessment tools for Phase 3

Assessment parameter	Median score	MDM	Level of consensus
Gait analysis (video/observation)	5	0.125	High
Pain	5	0.5	High
Leg length	5	0	High
Spinal posture	5	0.125	High
Muscle tone	5	0	High
Muscle power	5	0	High
Range of movement	5	0	High
Functional task performance	5	0.125	High
Patterns of movement	5	0.25	High
Gross motor function	4	0.75	Moderate
Psychosocial	4	0.75	Moderate

Abbreviation: MDM, mean deviation from median.

#### 3.1.7 Outcome measurement

In Phase 1, participants developed a list of 17 outcome measures used to evaluate episodes of care. Table 5 shows the high level of Phase 3 consensus (MDM range 0–0.375) for five individual tools measuring gait, muscle tone, ROM and motor function. Participants discussed the conflict between wanting to use appropriate tools and barriers to being able to use them, with P5 noting:

**TABLE 5:** The level of consensus on outcome measures for Phase 3

Outcome measure	Median score	MDM	Level of consensus
Passive range of motion	4	0.125	High
Modified Ashworth	5	0.375	High
Instrumented gait analysis	5	0.125	High
Gross Motor Function Measure (any)	4	0	High
Observational gait scale	4.5	0.375	High
Patient Reported Outcome Measures	3	0.625	Moderate
Modified Tardieu scale	3.5	0.75	Moderate
Therapy Outcome measures	3	0.75	Moderate
10‐m walk test	3.5	0.75	Moderate
Timed up and go	2.5	1.375	None
Edinburgh gait scale	2	1.875	None
Muscle power sprint test	2.5	1.625	None
Paediatric balance scale	3	1.375	None
6‐min walk test	3	0.875	None
Berg balance	3.5	1.625	None
Gross Motor Challenge Module	2.5	1.625	None
Quality Function Measure	3	1.375	None

Abbreviation: MDM, mean deviation from median.

Outcome measures used depend on time, space and equipment resources, as well as CYP compliance. (P5‐NG)

#### 3.1.8 Equipment advice, provision and referral

There was high consensus (MDM = 0.29) that physiotherapists usually provide mobility equipment and refer onto orthotic and wheelchair providers. There was high consensus (MDM = 0) that children with GMFCS Level III require a 24‐h postural management plan and assessment for alternate powered or wheelchair mobility to improve participation with school and leisure activities. Physiotherapists advocate for children to have choice about their mobility, with P3 noting that:

Wheelchair mobility [is] considered if it will improve independence and quality of life by improving access to community, reduce fatigue and pain levels. [We] want to encourage weight bearing and mobility but not at detriment to child's independence and participation. (P3‐S)

### 3.2 Physiotherapy interventions

In Phase 1, participants reached a high level of consensus on a list of interventions considered as usual care (Table 3). However, after consideration of the evidence summary (Table 1) presented alongside the survey, participants only reached consensus on including two of the six interventions into the usual care position statement (Table 6).

**TABLE 6:** The level of consensus on interventions included in the usual care position statement

Intervention	Median score	MDM	Level of consensus
Participation in sport and activity	5	0	High
Flexibility exercises	3	1	Low
Prolonged passive stretching	4	1.75	Low
Strength training	3	1.5	Low
Task‐specific training and functional activity	5	0.75	Moderate
Postural stability and balance exercises	3	1	Low

Abbreviation: MDM, mean deviation from median.

#### 3.2.1 Participation in sport and activity

There was high consensus (MDM = 0) that the physiotherapist's role is to encourage physical activities and facilitate children to access school and community resources to develop active lifestyles. Physiotherapists considered that the level of daily activity makes an important difference to the outcomes of children. They recognized that the level of support from home and school is critical, for example:

It is important that the child becomes part of the community and accesses local resources. It is part of a life‐long strategy. (P8‐S)

#### 3.2.2 Flexibility exercises

Physiotherapists described active flexibility exercises that move joints through full range, as usual care in Phase 1. Discussions concerning growth spurts frequently acknowledged that reduced ROM must be addressed in order to maintain the flexibility required for effective walking and balance. P5 noted that flexibility exercises are a:

useful adjunct in children who have stiff joints, MSK/postural asymmetry or who are tight due to growth spurts, to help to maintain ROM and flexibility, which helps with gait pattern, biomechanics and alignment. (P5‐S)

The literature review failed to find evidence that flexibility exercises improve balance and walking. Although three respondents wished to include this in usual care, there was low consensus (MDM = 0.86) in Phase 3.

#### 3.2.3 Prolonged passive stretching

In Phase 1, participants reached high consensus (MDM = 0) that prolonged passive stretching should be included in the list of usual care interventions. In Phase 3, there was low consensus (MDM = 0.86) that it should be included in the final position statement. The evidence summary focused on serial casting and standing frame use as being interventions provided by physiotherapists that deliver prolonged passive stretch. Prolonged passive stretching is more frequently provided using orthotics, a topic excluded in this study. There was divided opinion on inclusion between physiotherapists. Although the median score indicated that it should be included, there was low consensus on this. P5 explained how they use serial casting in individual cases, rather than as usual care:

Serial casting [may be used] on an individual basis e.g. to gain lost dorsiflexion, to enable an optimal AFO (Ankle‐foot orthoses) to be provided. (P5‐S)

P6 also described a more individual approach to using standing frames, in the presence of a specific risk:

I would only prescribe a standing frame for a child who is clearly at risk of developing knee flexion contractures, not as routine intervention. (P6‐S)

#### 3.2.4 Strength training

Strength training was identified as a key intervention in Phase 1. However, in Phase 3, there was low consensus on including this in usual care. The evidence summary highlighted the adverse events associated with this intervention and the lack of evidence that progressive resisted strength training improves walking and balance. Clinicians discussed integrating different exercise approaches that work through range of motion while working against resistance, for example, P3 reasoned:

Evidence is strong against the use of strengthening exercises. But is this because it was used in isolation, when in usual care we use a combination of different exercises/techniques to improve gait/balance. E.g., strengthening in addition to flexibility and range of movement in ankle/knee. (P3‐S)

#### 3.2.5 Task‐specific training and functional activity

There was moderate consensus that task‐specific training should be included in usual physiotherapy care. Task‐specific training within this context involves treadmill training, gait training and practising balance in functional situations. Participants' reservations over the availability of equipment such as treadmills influenced the consensus score, for example, P4:

Elements of task specific training should be included, when it can be performed at home and school environment. Not all Trusts have access to treadmill training so I would question whether this form of ‘task specific training’ is usual care. (P4‐S)

#### 3.2.6 Postural stability and balance exercises

There was strong consensus in Phase 1 that postural stability and balance activities are used to improve walking and balance. However, after consideration of the available evidence, there was low consensus on inclusion into usual care (MDM = 1). The literature review found evidence that supported the use of vibration plate training, which does not seem to be widely used in clinical practice, as voiced by P7:

I have not used full body vibration training so cannot comment on this type of therapy intervention. (P7‐S)

P5 talked about how they usually provide postural stability and balance exercise in a clinical setting:

[We] routinely provide postural stability and balance activities e.g. use of balance board. (P5‐S)

P2 was typical of the participants in expressing the way they combine approaches to include exercise targeting balance and posture:

Fun recreational activities are important for compliance and should be incorporated into daily life. Within these there will be elements of flexibility exercise, posture and balance. (P2‐S)

### 3.3 The impact of the COVID‐19 pandemic on usual care

The final phase of this study was carried out during the COVID‐19 pandemic, which may have influenced the results. The confirmatory survey was expanded to capture how usual care changed due to COVID‐19. All respondents reported the swift introduction of virtual appointments by video or telephone. These consultations had both positive and negative consequences, as outlined by P2:

This has not been ideal in terms of assessment of body function but has advantages for functional assessment [of children] in their own environment. (P2‐S)

Participants reported that assessments by virtual consultations were incomplete as they lacked manual assessment of movement quality, which affected clinical analysis and decision‐making. Some assessment and outcome measurement tools were not achievable during virtual consultations. Assessment of physical impairment was very limited, as explained by P3:

[We are] unable to ascertain strength/power/tone without hands‐on assessment or equipment, [we] can ask parents to measure range of movement but not as reliable as therapist due to angle of camera when carrying out virtual assessments. Parents have been able to send us videos of walking/other activities which has allowed us to compare side‐by‐side and review in slow motion to fully analyse. (P3‐S)

All participants said that essential face‐to‐face visits were possible for some children at home or at COVID‐secure premises.

Many respondents reported that they provided an assessment and management programme, but they were unable to offer routine monitoring or blocks of treatment at the height of the pandemic. The overall frequency and amount of contact per child has therefore reduced dramatically. All participants reported that children had reduced levels of activity in lockdown due to lack of access to sports facilities at school and in the community.

## 4 DISCUSSION

In this study, we explored ideas of what constitutes usual physiotherapy care to improve walking and balance for ambulant children with CP in the United Kingdom. The study used an NG consensus process. We examined the evidence supporting the interventions usually employed and developed a checklist of usual physiotherapy care for use in a future RCT (Appendix A).

We found a high level of consensus among physiotherapists to support the long‐term monitoring of children with CP at risk of musculoskeletal decline. This approach is backed by a growing evidence base that advocates routine surveillance of hip migration, joint range of motion and spinal posture for all children with CP (Cans, 2000). Where services do not currently include all children with CP in surveillance programmes, they give ‘red flag’ indicators for enabling timely access back into services. Physiotherapists play an essential role in identifying the need for orthotic and postural management equipment to optimize posture and mobility for children with CP.

Physiotherapists use collaborative goal setting to inform the need for treatment blocks usually delivered at an intensity of once per week, for 4–6 weeks. This contrasts with the frequency and intensity of usual physiotherapy care reported in some RCTs as 1–3 sessions of 30–60 min per week (Scholtes et al., 2007, 2012). Participants reached moderate consensus that task‐specific functional activity training should be included in usual care to improve balance and mobility. This is supported by both the National Institute of Clinical Excellence guidance (Mugglestone et al., 2012) and the evidence summary produced from the literature review. However, the reported frequency and intensity falls short of the dose reported to be effective in the literature. Intensive programmes delivered daily for 2 weeks have been shown to achieve the greatest functional improvements (Bleyenheuft et al., 2015). This level of resourcing for physiotherapy treatment programmes was not found within our study, which brings into question the ecological validity of these studies. Physiotherapy services in the United Kingdom might consider the efficiency gains of deploying current resources in a more concentrated way. Physiotherapists in our study applied the principles of research findings by integrating gait training in community, home and school activity programmes.

We found further divergence between the evidence and usual care delivered in the United Kingdom. Barriers to implementing evidence included lack of knowledge of new interventions such as vibration therapy. Additionally, physiotherapists reported lack of access to equipment such as body‐weight support treadmill and vibration plates. Our results show that there is a need for translation of research findings into clinical practice through dissemination of knowledge, appropriate resourcing and prioritising evidenced based interventions. Development of national clinical guidelines for paediatric physiotherapy may help to inform optimal use of precious resources.

Physiotherapy interventions for prolonged passive stretching alone were not considered usual care for all ambulant children. Physiotherapists consider the functional and social impact of using serial casting or standing frames with the child and caregivers and may choose to use them in individual cases. Physiotherapists have an important role in promoting independence and developing self‐advocacy in the children that they work with. Sometimes, the needs of the child might differ from those of the parents. For instance, some parents request that the focus of therapy should be on improving walking when the children with GMFCS III might find that wheeled mobility increases their levels of participation with peers. Physiotherapists were strident in promoting participation and emphasizing the voice of the child.

The main limitation to this study emerged during Phase 3 of the study. High levels of consensus on interventions were reached during Phases 1 and 2. During Phase 3, participants only reached consensus on two from the initial six interventions considered usual care. This may have been due to the smaller number of respondents in the final confirmatory survey. Furthermore, there was no opportunity at this stage for discussion of what participants understood by the evidence summary or newly emerged ideas, which possibly led to more variation in scoring and lower consensus. Participants in the study did not represent the whole of the United Kingdom, despite national advertising during the recruitment phase. This is a limitation as there may be wider variance from the consensus on usual care across and within the four countries. Another limiting factor of this study was that we only considered physiotherapy as delivered by physiotherapists. However, usual physiotherapy care programmes are delivered by parents and carers. Therefore, it is essential to measure this activity when measuring adherence to usual care in a trial control group.

In 2020, when the study was carried out, the COVID‐19 pandemic hugely influenced the provision of usual care for ambulant children with CP. School closure resulted in lack of access to therapeutic classroom support and equipment. It is likely that many parents and guardians were unable to replicate therapy provision at home due to work, other care responsibilities or their own health needs. Children had difficulty accessing usual recreational activities during lockdown and shielding. Although the full effect of this pandemic on services for children has yet to be evaluated, this study was able to capture the initial adaptations in the delivery of usual care.

This study used a modified NGT consensus process to develop a position statement and checklist of usual physiotherapy care aimed at improving walking and balance in children with CP in the United Kingdom. It is important for RCTs to define the usual care carried out in a control group to measure the effectiveness of a novel intervention. We found that physiotherapists combine heterogeneous approaches and create tailor‐made programmes to meet the needs of individual children and families. The frequency and intensity of physiotherapy interventions falls short of dosage reported to be effective in the literature.

## 5 CONCLUSION

Physiotherapists reached consensus on two usual care interventions and a checklist was developed to inform future RCTs. Further consensus work is required to establish clinical guidelines to standardize usual physiotherapy care in the United Kingdom. This study is a first step towards defining physiotherapy care effective at improving balance and walking for ambulant children with CP in the United Kingdom.

## Data Availability

The data that support the findings of this study are available from the corresponding author upon reasonable request.

## APPENDIX A CHECKLIST OF USUAL PHYSIOTHERAPY CARE AIMED AT IMPROVING WALKING AND BALANCE FOR AMBULANT CHILDREN WHO HAVE CEREBRAL PALSY

Please enter an X in each box to indicate criteria are met.

**Table:**

1	Referral and discharge criteria	
**a**	Children and young people with GMFCS III are managed on a long‐term multidisciplinary care pathway from initial referral to transition into adult services.	
**b**	Children with GMFCS Levels I and II are offered episodes of care related to their functional needs and are discharged where there are no identifiable needs or their musculoskeletal condition is stable.	
**c**	Where children are discharged, information is given to them, and their carers to help them identify key triggers/red flags for timely re‐referral into the service.	
**d**	Ambulant children have ongoing access to orthotics as required.	

2	Location of physiotherapy	
**a**	Children are offered virtual consultations and face‐to‐face appointments, as appropriate.	
**b**	Appointments take place in a children's physiotherapy department or a child‐friendly general outpatient clinic setting.	
**c**	Appointments are offered in school or at home when indicated due to environmental needs or comorbidities.	

3	AssessmentThe following core areas should be included in assessment:	
**a**	Gait analysis (video/observation)	
**b**	Pain	
**c**	Leg length	
**d**	Spinal posture	
**e**	Muscle tone	
**f**	Muscle power	
**g**	Range of movement	
**h**	Functional task performance	
**i**	Balance	
**j**	Patterns of movement	
**k**	Gross motor function	
**l**	Psychosocial	

4	Goal setting	
	Specific Measureable Achievable Realistic Timed (SMART) goal are set collaboratively with the child and their family.	

5	Physiotherapy interventions	
**a**	Functional, task‐specific training is used to treat walking and balance difficulties. Adjuncts to task‐specific training include using a treadmill with or without body‐weight support.	
**b**	Vibration training is used to improve balance and posture (where equipment exists).	
**c**	Physiotherapists encourage and facilitate children to develop active lifestyles including aerobic exercise, fitness training and modified sport.	
**d**	Strength training using progressive resisted exercise is not employed as a treatment to improve walking and balance.	
**e**	Prolonged passive stretching is used to manage contractures using orthotics, serial casting and supported standing programmes.	
**f**	Postural management approaches are employed for children with GMFCS III, including mobility equipment and environmental adaptation.	
**g**	Exercise and functional activities that encourage full joint range are recommended where there is risk of contracture development.	

6	Frequency and intensity of physiotherapy	
**a**	The frequency of blocks and reviews is determined by clinical need.	
**b**	Blocks of 4–6 appointments are offered where there is a functional need.	
**c**	Children are reviewed every 3–12 months.	

7	Outcome measurements The following outcome measures are considered for the evaluation of interventions aimed at improving balance and walking:	
**a**	Passive range of movement	
**b**	Modified Ashworth scale	
**c**	Modified Tardieu scale	
**d**	MRC (Oxford) scale muscle strength	
**e**	Gross Motor Function Measure	
**f**	Observational Gait Scale	
**g**	10‐m walk test	
**h**	Instrumented gait analysis	
**i**	Patient reported outcome measures, e.g. Goal Attainment Scale	
**j**	Therapy Outcome Measures	

8	Advice and information	
**a**	Support is given to the child, parents and school to understand the impact of diagnosis and prognosis of the child's condition.	
**b**	Signposting to local and national resources such as support groups, local offer and charitable organizations.	
**c**	Training and education is offered for child, parents, school and other health and social care professionals.	
**d**	Timely input to SEND/EHCP process.	
**e**	Where the family or young person gives consent, information is shared with education, health and social care services through reports, therapy advice/programmes and clinic letters.	

## References

[cch12977-bib-0001] Allen, J. , Dyas, J. , & Jones, M. (2004). Building consensus in health care: A guide to using the nominal group technique. British Journal of Community Nursing, 9(3), 110–114. 10.12968/bjcn.2004.9.3.12432 15028996

[cch12977-bib-0002] Anttila, H. , Autti‐Rämö, I. , Suoranta, J. , Mäkelä, M. , & Malmivaara, A. (2008). Effectiveness of physical therapy interventions for children with cerebral palsy: A systematic review. BMC Pediatrics, 8, 14. 10.1186/1471-2431-8-14 18435840 PMC2390545

[cch12977-bib-0003] Balshem, H. , Helfand, M. , Schünemann, H. J. , Oxman, A. D. , Kunz, R. , Brozek, J. , Vist, G. E. , Falck‐Ytter, Y. , Meerpohl, J. , & Norris, S. (2011). GRADE guidelines: 3. Rating the quality of evidence. Journal of Clinical Epidemiology, 64(4), 401–406. 10.1016/j.jclinepi.2010.07.015 21208779

[cch12977-bib-0004] Bania, T. , Chiu, H.‐C. , & Billis, E. (2019). Activity training on the ground in children with cerebral palsy: Systematic review and meta‐analysis. Physiotherapy Theory and Practice, 35(9), 810–821. 10.1080/09593985.2018.1460647 29659303

[cch12977-bib-0005] Bartlett, D. J. , & Palisano, R. J. (2002). Physical therapists' perceptions of factors influencing the acquisition of motor abilities of children with cerebral palsy: Implications for clinical reasoning. Physical Therapy, 82(3), 237–248. 10.1093/ptj/82.3.237 11869152

[cch12977-bib-0006] Bleyenheuft, Y. , Arnould, C. , Brandao, M. B. , Bleyenheuft, C. , & Gordon, A. M. (2015). Hand and arm bimanual intensive therapy including lower extremity (HABIT‐ILE) in children with unilateral spastic cerebral palsy: A randomized trial. Neurorehabilitation and Neural Repair, 29(7), 645–657. 10.1177/1545968314562109 25527487

[cch12977-bib-0007] Booth, A. T. C. , Buizer, A. I. , Meyns, P. , Oude Lansink, I. L. B. , Steenbrink, F. , & van der Krogt, M. M. (2018). The efficacy of functional gait training in children and young adults with cerebral palsy: A systematic review and meta‐analysis. Developmental Medicine and Child Neurology, 60(9), 866–883. 10.1111/dmcn.13708 29512110

[cch12977-bib-0008] Cans, C. (2000). Surveillance of cerebral palsy in Europe: a collaboration of cerebral palsy surveys and registers. Developmental Medicine & Child Neurology, 42(12), 816–824. 10.1111/j.1469-8749.2000.tb00695.x 11132255

[cch12977-bib-0009] Centre for Evidence‐Based Medicine, OCEBM Levels of Evidence Working Group . (2011). The Oxford 2011 levels of evidence.

[cch12977-bib-0010] Clutterbuck, G. , Auld, M. , & Johnston, L. (2019). Active exercise interventions improve gross motor function of ambulant/semi‐ambulant children with cerebral palsy: A systematic review. Disability and Rehabilitation, 41(10), 1131–1151. 10.1080/09638288.2017.1422035 29303007

[cch12977-bib-0011] Corsi, C. , Santos, M. M. , Moreira, R. F. C. , dos Santos, A. N. , de Campos, A. C. , Galli, M. , & Rocha, N. A. C. F. (2021). Effect of physical therapy interventions on spatiotemporal gait parameters in children with cerebral palsy: a systematic review. Disability and Rehabilitation, 43(11), 1507–1516. 10.1080/09638288.2019.1671500 31588810

[cch12977-bib-0018] Delbecq, A. L. , Van de Ven, A. H. , & Gustafson, D. H. (1975). Group techniques for program planning: A guide to nominal group and Delphi processes. Scott Foresman. http://eduq.info/xmlui/handle/11515/11368

[cch12977-bib-0012] Dewar, R. , Love, S. , & Johnston, L. M. (2015). Exercise interventions improve postural control in children with cerebral palsy: A systematic review. Developmental Medicine & Child Neurology, 57(6), 504–520. 10.1111/dmcn.12660 25523410

[cch12977-bib-0013] Elnahhas, A. M. , Elshennawy, S. , & Aly, M. G. (2019). Effects of backward gait training on balance, gross motor function, and gait in children with cerebral palsy: A systematic review. Clinical Rehabilitation, 33(1), 3–12. 10.1177/0269215518790053 30043634

[cch12977-bib-0014] Franki, I. , Desloovere, K. , Cat, J. , Feys, H. , Molenaers, G. , Calders, P. , Vanderstraeten, G. , Himpens, E. , & Broeck, C. (2012). The evidence‐base for basic physical therapy techniques targeting lower limb function in children with cerebral palsy: A systematic review using the International Classification of Functioning, Disability and Health as a conceptual framework. Journal of Rehabilitation Medicine, 44(5), 385–395. 10.2340/16501977-0983 22549646

[cch12977-bib-0016] Galey, S. A. , Lerner, Z. F. , Bulea, T. C. , Zimbler, S. , & Damiano, D. L. (2017). Effectiveness of surgical and non‐surgical management of crouch gait in cerebral palsy: A systematic review. Gait & Posture, 54, 93–105. 10.1016/j.gaitpost.2017.02.024 28279852 PMC9619302

[cch12977-bib-0017] Gunn, H. , Endacott, R. , Haas, B. , Marsden, J. , & Freeman, J. (2018). Development of a balance, safe mobility and falls management programme for people with multiple sclerosis. Disability and Rehabilitation, 40(24), 2857–2866. 10.1080/09638288.2017.1362041 28783979

[cch12977-bib-0019] Hägglund, G. , Alriksson‐Schmidt, A. , Lauge‐Pedersen, H. , Rodby‐Bousquet, E. , Wagner, P. , & Westbom, L. (2014). Prevention of dislocation of the hip in children with cerebral palsy: 20‐year results of a population‐based prevention programme. The Bone & Joint Journal, 96(11), 1546–1552. 10.1302/0301-620X.96B11.34385 25371472

[cch12977-bib-0020] Hanna, S. E. , Bartlett, D. J. , Rivard, L. M. , & Russell, D. J. (2008). Reference curves for the gross motor function measure: Percentiles for clinical description and tracking over time among children with cerebral palsy. Physical Therapy, 88(5), 596–607. 10.2522/ptj.20070314 18339799 PMC2390723

[cch12977-bib-0021] Hannes, K. , Lockwood, C. , & Pearson, A. (2010). A comparative analysis of three online appraisal instruments' ability to assess validity in qualitative research. Qualitative Health Research, 20(12), 1736–1743. 10.1177/1049732310378656 20671302

[cch12977-bib-0022] Harvey, N. , & Holmes, C. A. (2012). Nominal group technique: An effective method for obtaining group consensus. International Journal of Nursing Practice, 18(2), 188–194. 10.1111/j.1440-172X.2012.02017.x 22435983

[cch12977-bib-0023] Kamp, F. A. , Lennon, N. , Holmes, L. , Dallmeijer, A. J. , Henley, J. , & Miller, F. (2014). Energy cost of walking in children with spastic cerebral palsy: Relationship with age, body composition and mobility capacity. Gait & Posture, 40(1), 209–214. 10.1016/j.gaitpost.2014.03.187 24768085

[cch12977-bib-0024] Kimoto, M. , Yonetsu, R. , Okada, K. , Horioka, W. , Kondou, T. , Sasaki, M. , & Sakamoto, H. (2019). Effect of home‐based training focused on increasing maximum step length in walking function of children with cerebral palsy. Physical Therapy Reviews, 24(6), 358–365. 10.1080/10833196.2019.1664083

[cch12977-bib-0025] Moher, D. , Altman, D. G. , Liberati, A. , & Tetzlaff, J. (2011). PRISMA statement. Epidemiology, 22(1), 128. 10.1097/EDE.0b013e3181fe7825 21150360

[cch12977-bib-0026] Moreau, N. G. , Bodkin, A. W. , Bjornson, K. , Hobbs, A. , Soileau, M. , & Lahasky, K. (2016). Effectiveness of rehabilitation interventions to improve gait speed in children with cerebral palsy: Systematic review and meta‐analysis. Physical Therapy, 96(12), 1938–1954. 10.2522/ptj.20150401 27313240 PMC5131187

[cch12977-bib-0027] Morris, C. , Simkiss, D. , Busk, M. , Morris, M. , Allard, A. , Denness, J. , Janssens, A. , Stimson, A. , Coghill, J. , Robinson, K. , Fenton, M. , & Cowan, K. (2015). Setting research priorities to improve the health of children and young people with neurodisability: A British Academy of Childhood Disability‐James Lind Alliance research priority setting partnership. BMJ Open, 5(1), e006233. 10.1136/bmjopen-2014-006233 PMC4316435 25631309

[cch12977-bib-0028] Mugglestone, M. A. , Eunson, P. , & Murphy, M. S. (2012). Spasticity in children and young people with non‐progressive brain disorders: summary of NICE guidance. BMJ, 345, e4845. 10.1136/bmj.e4845 22836108

[cch12977-bib-0030] Novak, I. , Mcintyre, S. , Morgan, C. , Campbell, L. , Dark, L. , Morton, N. , Stumbles, E. , Wilson, S. A. , & Goldsmith, S. (2013). A systematic review of interventions for children with cerebral palsy: State of the evidence. Developmental Medicine & Child Neurology, 55(10), 885–910. 10.1111/dmcn.12246 23962350

[cch12977-bib-0031] Novak, I. , Morgan, C. , Fahey, M. , Finch‐Edmondson, M. , Galea, C. , Hines, A. , Langdon, K. , Namara, M. M. , Paton, M. C. B. , Popat, H. , Shore, B. , Khamis, A. , Stanton, E. , Finemore, O. P. , Tricks, A. , te Velde, A. , Dark, L. , Morton, N. , & Badawi, N. (2020). State of the evidence traffic lights 2019: Systematic review of interventions for preventing and treating children with cerebral palsy. Current Neurology and Neuroscience Reports, 20(2), 3. 10.1007/s11910-020-1022-z 32086598 PMC7035308

[cch12977-bib-0032] Oskoui, M. , Coutinho, F. , Dykeman, J. , Jetté, N. , & Pringsheim, T. (2013). An update on the prevalence of cerebral palsy: A systematic review and meta‐analysis. Developmental Medicine & Child Neurology, 55(6), 509–519. 10.1111/dmcn.12080 23346889

[cch12977-bib-0033] Palisano, R. , Rosenbaum, P. , Walter, S. , Russell, D. , Wood, E. , & Galuppi, B. (1997). Gross motor function classification system for cerebral palsy. Developmental Medicine and Child Neurology, 39(4), 214–223. 10.1111/j.1469-8749.1997.tb07414.x 9183258

[cch12977-bib-0034] Palisano, R. J. , Copeland, W. P. , & Galuppi, B. E. (2007). Performance of physical activities by adolescents with cerebral palsy. Physical Therapy, 87(1), 77–87. 10.2522/ptj.20060089 17179440

[cch12977-bib-0035] Potter, M. , Gordon, S. , & Hamer, P. (2004). The nominal group technique: A useful consensus methodology in physiotherapy research. New Zealand Journal of Physiotherapy, 32(2), 70–75.

[cch12977-bib-0036] Ritzmann, R. , Stark, C. , & Krause, A. (2018). Vibration therapy in patients with cerebral palsy: A systematic review. Neuropsychiatric Disease and Treatment, 14, 1607–1625. 10.2147/NDT.S152543 29950843 PMC6018484

[cch12977-bib-0037] Rosenbaum, P. F. , Paneth, N. , Leviton, A. , Goldstein, M. , Bax, M. , Damiano, D. , Dan, B. , & Jacobsson, B. (2007). A report: The definition and classification of cerebral palsy April 2006. Developmental Medicine and Child Neurology. Supplement, 109, 8–14. 17370477

[cch12977-bib-0038] Royal College of Physicians . (2016). National clinical guideline for stroke ‐ SSNAP (5th ed.). Royal College of Physicians.

[cch12977-bib-0039] Salem, Y. , Lovelace‐Chandler, V. , Zabel, R. J. , & McMillan, A. G. (2010). Effects of prolonged standing on gait in children with spastic cerebral palsy. Physical & Occupational Therapy in Pediatrics, 30(1), 54–65. 10.3109/01942630903297177 20170432

[cch12977-bib-0040] Scholtes, V. A. , Becher, J. G. , Janssen‐Potten, Y. J. , Dekkers, H. , Smallenbroek, L. , & Dallmeijer, A. J. (2012). Effectiveness of functional progressive resistance exercise training on walking ability in children with cerebral palsy: A randomized controlled trial. Research in Developmental Disabilities, 33(1), 181–188. 10.1016/j.ridd.2011.08.026 22093663

[cch12977-bib-0041] Scholtes, V. A. , Dallmeijer, A. J. , Knol, D. L. , Speth, L. A. , Maathuis, C. G. , Jongerius, P. H. , & Becher, J. G. (2007). Effect of multilevel botulinum toxin A and comprehensive rehabilitation on gait in cerebral palsy. Pediatric Neurology, 36(1), 30–39. 10.1016/j.pediatrneurol.2006.09.010 17162194

[cch12977-bib-0042] Tustin, K. , & Patel, A. (2017). A critical evaluation of the updated evidence for casting for equinus deformity in children with cerebral palsy. Physiotherapy Research International, 22(1), e1646. 10.1002/pri.1646 26351821

[cch12977-bib-0043] Tutt, R. , & Williams, P. (2015). The SEND code of practice 0–25 years: Policy, provision and practice. Sage.

[cch12977-bib-0044] Valentín‐Gudiol, M. , Mattern‐Baxter, K. , Girabent‐Farrés, M. , Bagur‐Calafat, C. , Hadders‐Algra, M. , & Angulo‐Barroso, R. M. (2017). Treadmill interventions in children under six years of age at risk of neuromotor delay. Cochrane Database of Systematic Reviews, 2017(7), 1465–1858. 10.1002/14651858.cd009242.pub3 PMC6483121 28755534

[cch12977-bib-0045] Ven, A. H. V. D. , & Delbecq, A. L. (1974). The effectiveness of nominal, Delphi, and interacting group decision making processes. Academy of Management Journal, 17(4), 605–621. 10.5465/255641

[cch12977-bib-0046] Wordie, S. J. , Robb, J. E. , Hägglund, G. , Bugler, K. E. , & Gaston, M. S. (2020). Hip displacement and dislocation in a total population of children with cerebral palsy in Scotland: Status after five years' hip surveillance. The Bone & Joint Journal, 102(3), 383–387. 10.1302/0301-620X.102B3.BJJ-2019-1203.R1 32114804

[cch12977-bib-0047] World Health Organization . (2007). International classification of functioning, disability, and health: Children & youth version: ICF‐CY. World Health Organization.

